# Comparative transcriptomics analysis reveals defense mechanisms of *Manihot esculenta* Crantz against *Sri Lanka Cassava MosaicVirus*

**DOI:** 10.1186/s12864-024-10315-0

**Published:** 2024-05-02

**Authors:** Somruthai Chaowongdee, Nattachai Vannatim, Srihunsa Malichan, Nattakorn Kuncharoen, Pumipat Tongyoo, Wanwisa Siriwan

**Affiliations:** 1 Center of Excellence on Agricultural Biotechnology (AG-BIO/MHESI), Bangkok, 10900 Thailand; 2 Center for Agricultural Biotechnology, Kasetsart University, Kamphaengsaen Campus, Nakhon Pathom, 73140 Thailand; 3 Department of Plant Pathology, Faculty of Agriculture, Kasetsart University, Bangkok, 10900 Thailand

**Keywords:** Transcriptomics, Cassava mosaic disease, Tolerant and susceptible phenotypes, Gene regulation, Plant defense

## Abstract

**Background:**

Cassava mosaic disease (CMD), caused by *Sri Lankan cassava mosaic virus* (SLCMV) infection, has been identified as a major pernicious disease in *Manihot esculenta* Crantz (cassava) plantations. It is widespread in Southeast Asia, especially in Thailand, which is one of the main cassava supplier countries. With the aim of restricting the spread of SLCMV, we explored the gene expression of a tolerant cassava cultivar vs. a susceptible cassava cultivar from the perspective of transcriptional regulation and the mechanisms underlying plant immunity and adaptation.

**Results:**

Transcriptomic analysis of SLCMV-infected tolerant (Kasetsart 50 [KU 50]) and susceptible (Rayong 11 [R 11]) cultivars at three infection stages—that is, at 21 days post-inoculation (dpi) (early/asymptomatic), 32 dpi (middle/recovery), and 67 dpi (late infection/late recovery)—identified 55,699 expressed genes. Differentially expressed genes (DEGs) between SLCMV-infected KU 50 and R 11 cultivars at (i) 21 dpi to 32 dpi (the early to middle stage), and (ii) 32 dpi to 67 dpi (the middle stage to late stage) were then identified and validated by real-time quantitative PCR (RT-qPCR). DEGs among different infection stages represent genes that respond to and regulate the viral infection during specific stages. The transcriptomic comparison between the tolerant and susceptible cultivars highlighted the role of gene expression regulation in tolerant and susceptible phenotypes.

**Conclusions:**

This study identified genes involved in epigenetic modification, transcription and transcription factor activities, plant defense and oxidative stress response, gene expression, hormone- and metabolite-related pathways, and translation and translational initiation activities, particularly in KU 50 which represented the tolerant cultivar in this study.

**Supplementary Information:**

The online version contains supplementary material available at 10.1186/s12864-024-10315-0.

## Background

Cassava (*Manihot esculenta* Crantz) is one of the most important carbohydrate plants in the world. It has various uses, including human and animal consumption and plant-based energy production, and has a high carbohydrate yield, like rice and maize [1]. It has been estimated that more than 800 million people globally consume cassava as their main food crop, especially in Africa and Latin America [2].

A major problem for cassava cultivation in Southeast Asia is cassava mosaic disease (CMD) [3]. CMD is caused by cassava mosaic viruses, which belong to the genus *Begomovirus* in the family *Geminiviridae*. Cassava mosaic viruses are twinned-particle viruses, with two circular, single-stranded DNA components (DNA-A and DNA-B) [4]. Globally, there are 11 species of cassava mosaic viruses [5, 6]. However, in Asia, there are only two major species, *Sri Lankan cassava mosaic mirus* (SLCMV) and *Indian cassava mosaic virus* (ICMV) [7], with the former being the most prominent in Southeast Asia [3].

In general, SLCMV is transmitted via infected whiteflies (*Bemisia tabaci*). The typical symptoms of CMD are foliage with chlorotic, mosaic, mottled patterns, distorted and crumpled leaves, reduced leaflet size, and stunting [8]. The first SLCMV outbreak in Southeast Asia occurred in Cambodia in 2015 [9]. CMD was first reported in Thailand in 2018 [10], and the Department of Agriculture Extension in Thailand reported that the outbreak involved approximately 51,200 hectares, covering 17 provinces [11].

Owing to the adaptation of cassava to SLCMV and the resultant development of various cultivars with distinct phenotypes, resistant, tolerant, and susceptible cultivars of cassava have been developed. A resistant cultivar has *R* gene resistance [12–14]; a tolerant cultivar adapts to the virus and has reduced symptoms (recovery symptoms) on new leaves, by decreasing the virus titer [12, 14–16]; and a susceptible cultivar is unable to avoid pathogen infection [14].

Transcriptomics analysis represents a powerful and essential tool for the new era of biological and breeding studies of plants. It can be used to identify variations in transcription across different plant–virus interactions, providing information on plant adaptations, including plant defense mechanisms [17, 18]. Freeborough et al. (2021) [19] and Allie et al. (2014) [20] studied *South African cassava mosaic virus* (SACMV) transcriptional reprogramming after its infection of tolerant and susceptible cassava cultivars. The results provide information on the gene–protein networks and differential gene expression during infection of tolerant vs. susceptible cassava cultivars, with the eventual development of gene sets that differed between these two cultivars. Understanding CMD severity and the mechanisms underlying the diseases caused by SLCMV infection is essential to help clarify the pathogen–host interactions and direct future research to further understand plant defenses against this virus and the recovery mechanisms after infection.

This study involved a transcriptomics analysis of SLCMV-infected tolerant and susceptible cassava cultivars at 21 days post-inoculation (dpi) (early/asymptomatic), 32 dpi (middle/recovery), and 67 dpi (late infection/late recovery) [1, 14, 19–21]. Profiles of differentially expressed genes (DEGs) based on next-generation sequencing of tolerant and susceptible cassava cultivars were compared to gain insights into the antiviral mechanisms, including post-transcriptional gene silencing (PTGS) and transcriptional gene silencing (TGS). We hypothesized that the gene expression data might provide information on the plant defense mechanisms triggered by SLCMV, including the mechanisms that lead to recovery or susceptibility symptoms. The DEGs among distinct infection stages indicate the mechanisms related to the cassava–SLCMV relationship during the different infection stages.

## Results and discussion

### Comparison of symptoms in SLCMV-infected KU 50 vs. R 11

SLCMV inoculation was performed using a grafting technique. According to PCR with *AC1*-specific primers, all samples were positive for SLCMV at 21, 32, and 67 dpi, which was consistent with the appearance of CMD symptoms in the plants.

**Fig. 1:**
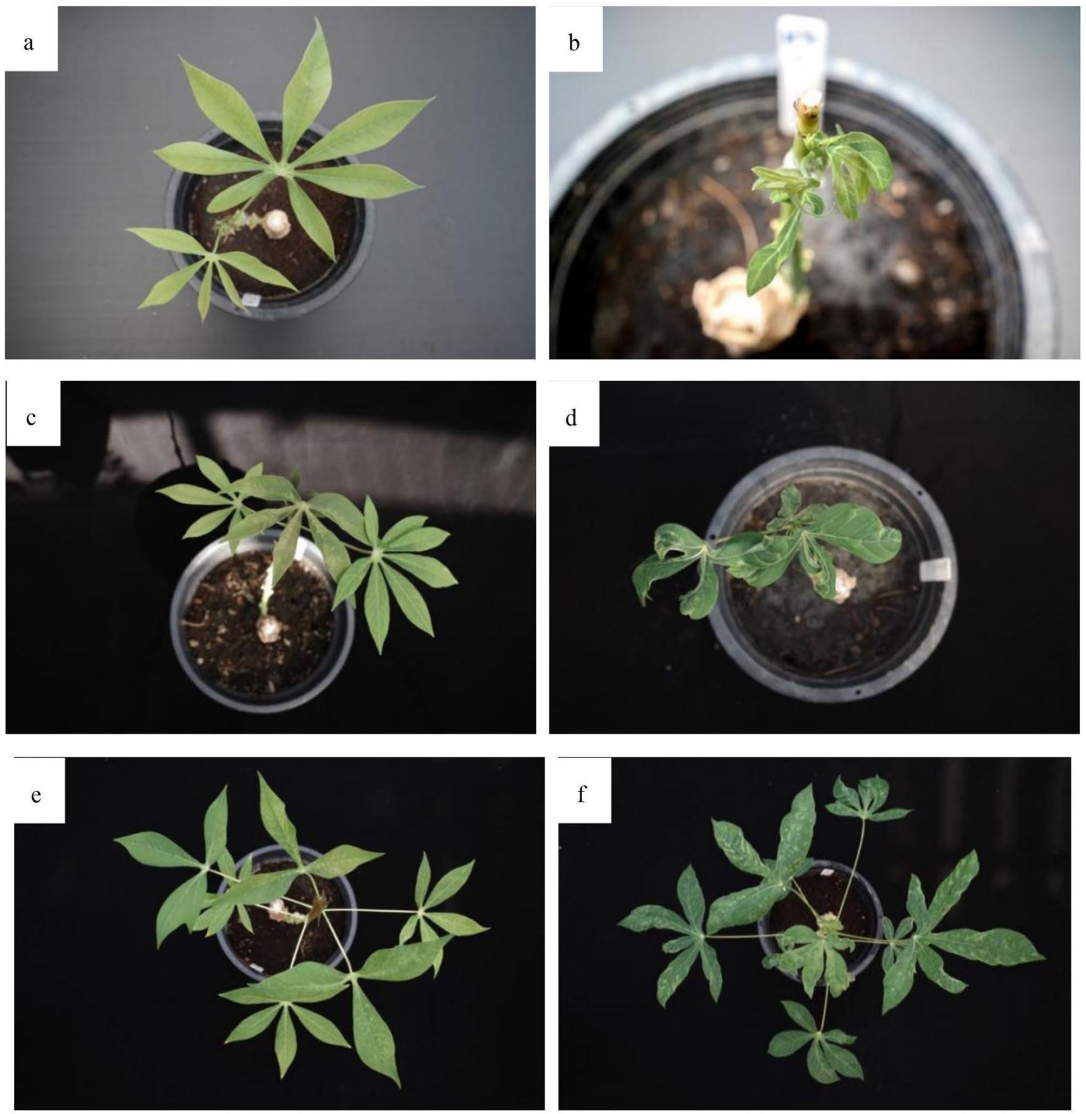
Symptoms of cassava mosaic disease in SLCMV-infected leaves (SLCMV inoculation was performed using a grafting technique) (**a**) KU 50 and (**b**) R 11 cultivars of cassava leaves at 21 days post-inoculation (dpi), (**c**) KU 50 and (**d**) R 11 at 32 dpi, and (**e**) KU 50 and (**f**) R 11 at 67 dpi

At 21 dpi, there was disordered and reduced vein development in young R 11 leaves, particularly regarding the apical leaves; however, in emerging and young KU 50 leaves, no obvious symptoms were visible (Fig. 1a–b). At 32 dpi, distinct symptoms (decreased leaf size, disordered veins, pale leaves, and mosaic symptoms) were observed in R 11 leaves, and there was decreased leaf size and mild mosaic symptoms in young KU 50 leaves (Fig. 1c–d). At 67 dpi, there were moderate mosaic symptoms in young KU 50 leaves, whereas older leaves exhibited recovery, with milder symptoms. In contrast, R 11 continued to exhibit severe symptoms at this time point (Fig. 1e–f). Despite the differences in cassava cultivars, the symptoms were consistent with those reported by Pierce and Ray (2013) [22] and Fofana et al. (2004) [23], who found that *African cassava mosaic virus* (ACMV) infection symptoms began to appear on the entire surface of the expanded leaves at 21 dpi. Freeborough et al. (2021) [19] and Aille et al. (2014) [20] suggested that 32 dpi represented a middle or recovery stage of ACMV infection, while 67 dpi represented a late stage of infection (with similar symptoms as at 32 dpi). Although the species of cassava mosaic virus used in our study (SLCMV) was different from that used in previous research (ACMV), our results indicated that the periods of symptom development and the overall duration of infection were similar in the various studies.

The hallmark of cassava cultivar KU 50 as being CMD tolerant is widely recognized across the Southeast Asia subcontinent [24]. Research by Ntui et al. (2015) [25] demonstrated this remarkable feature in the KU 50 cultivar when evaluating the capacity to build the tolerant phenotype under SLCMV infection. Their experiments advocated that the interfering dsRNA elements during the transcriptional process, the SLCMV transcribed element was triggered the target mRNA interfering within the KU 50 itself as mentioned as siRNA and led to specific degradation through the phenomenal defense mechanism concept called RNA silencing. This process resulted in restricted viral replication and reduced viral accumulation, including a decrease in disease symptoms, thus demonstrating that the production of specific siRNA derived from the RNA silencing was linked to resistance, reversion, and PTGS. Malik et al. (2022) [26] reinforced this view, reporting that the KU 50 cultivar consistently showed fewer symptoms associated with the CMD tolerance phenotype in field experiments exploring disease severity in KU 50 and R 11 cultivars under the SLCMV-infected condition. KU 50 displayed the fewest symptoms, whereas R 11 had the highest score for symptoms. Furthermore, the two cultivars exhibited a significant difference in disease incidence, which was determined from the percentage of infected plants and area under disease progress curves (AUDPC)—R 11 had the highest values, whereas KU 50 consistently ranked as lowest for both parameters.

### Genes expressed in KU 50 and R 11 at the three time points

The RNA-seq data were aligned and mapped to the *Manihot esculenta* reference sequence available in the National Center for Biotechnology Information (NCBI) database. The raw sequence reads were deposited in the NCBI database under the Sequence Read Archive accession number PRJNA1040252. There were 55,699 expressed genes in total across all the samples (Additional file 2).

**Fig. 2:**
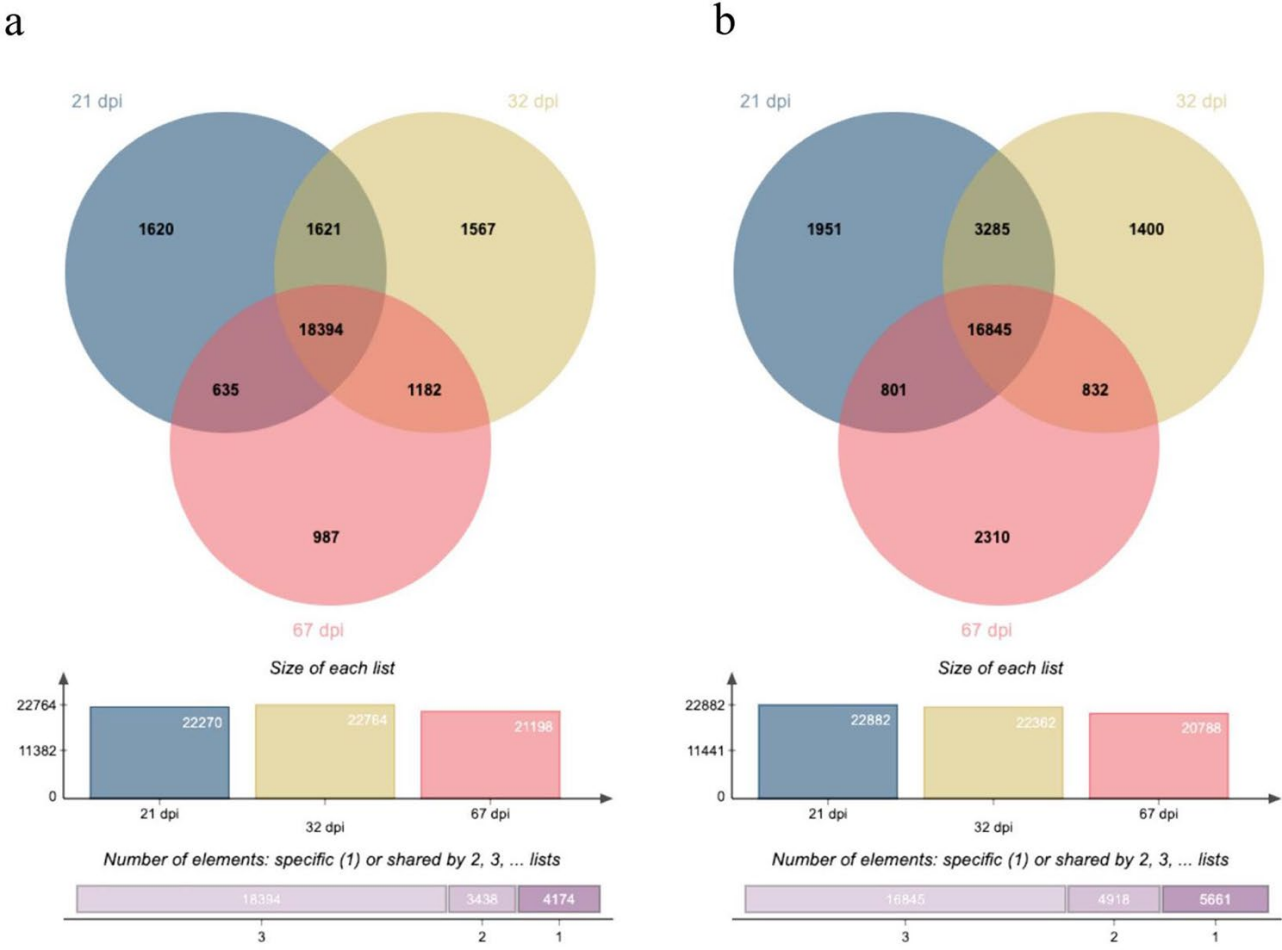
Venn diagrams of all transcripts or genes in SLCMV-infected (**a**) KU 50 and (**b**) R 11 cultivars at 21, 32, and 67 dpi

Venn diagrams were generated to visually compare the time points in KU 50 (Fig. 2a) and R 11 (Fig. 2b) in terms of numbers of genes. In KU 50, 18,394 genes were expressed at all three time points, 1,620 genes solely at 21 dpi, 1,567 genes solely at 32 dpi, 987 genes solely at 67 dpi, 1,621 genes at both 21 and 32 dpi, 1,182 genes at both 32 and 67 dpi, and 635 genes at both 21 and 67 dpi (Fig. 2a). In R 11, 16,845 genes were expressed at all three time points, 1,951 genes solely at 21 dpi, 1,400 genes solely at 32 dpi, 2,310 genes solely at 67 dpi, 3,285 genes at both 21 and 32 dpi, 832 genes at both 32 and 67 dpi, and 801 genes at both 21 and 67 dpi (Fig. 2b).

Some of the identified genes and their functions, determined from Gene Ontology (GO) term examination, are displayed in Table 1, which is divided as detected time points and cultivar. Following further analysis, Table 2 shows the DEGs associated with six GO categories that are relevant to plant immunity against viral infection: (1) epigenetic modification, (2) transcription and transcription factor (TF) activities, (3) plant defense and oxidative stress response, (4) gene expression, (5) hormone- and metabolite-related activities, and (6) translation and translational initiation. Plants have developed various defense mechanisms, including pathogen-associated molecular pattern (PAMP)-triggered immunity (PTI) and effector-triggered immunity (ETI) pathways, which activate a complex regulatory network, hormone signal transduction, and transcriptional reprograming. As indicated in Table 1, recent research [27, 28] shows that epigenetic factors play a key role in transcriptional reprogramming and modulate plant immune responses. Thus, epigenetic mechanisms (such as DNA methylation and histone modification) are crucial in the various regulatory mechanisms of plant defense responses.

**Table 1:** Expressed genes and their functions in SLCMV-infected KU 50 and R 11 cultivars at 21-, 32-, and 67 dpi

RefSeq NCBI accession ID	Gene stable ID	GO term name*	KU50	R11	
			DPI		
21 dpi					
XM_021740451.1	MANES_16G043500	DNA binding and templated transcription including binding transcription factor activity and brassinosteroid mediated signaling pathway	21	21, 32	
XM_021750310.1	MANES_03G080900	DNA binding and templated transcription including RNA polymerase binding and transcription factor activity	21, 32	ND**	
XM_021755496.1	MANES_04G105000	DNA binding and templated transcription including binding transcription factor activity	21	21, 32	
XM_021755538.1	MANES_04G080300	Response to auxin	21	ND	
XM_021737402.1	MANES_14G151500	Cellular response to salicylic acid stimulus and regulation of salicylic acid mediated signaling pathway.	21, 32, 67	21, 32, 67	
XM_021755704.1	MANES_04G052000	Ethylene-activated signaling pathway	21, 32, 67	21, 32, 67	
XM_021762771.1	MANES_07G056100	Pyruvate kinase activity	21, 32, 67	21, 32	
XM_021737402.1	MANES_14G151500	Regulation of defense response	21, 32, 67	21, 32, 67	
XM_021776058.1	MANES_13G113000	Regulation of gene expression	21	ND	
XM_021779209.1	MANES_01G114900	Transferase and methionyl-tRNA formyltransferase activity, translation, translation and translational initiation	21, 32, 67	21	
XM_021747204.1	MANES_S076200	Cell redox homeostasis and protein-disulfide reductase activity	21	ND	
XM_021745301.1	MANES_18G051100	Cation transmembrane transport	21, 32, 67	21	
XM_021751035.1	MANES_03G034300	Integral component of membrane	ND	21	
XM_021761247.1	MANES_06G119600	Ubiquitin-like modifier activating enzyme activity	ND	21	
XM_021759639.1	MANES_06G165400 and MANES_06G165300	1-acylglycerol-3-phosphate O-acyltransferase activity and integral component of membrane	21	ND	
XM_021776226.1	MANES_13G149700	Integral component of membrane	ND	21	
XM_021743397.1	MANES_02G096700	Integral component of membrane	ND	21	
XM_021762311.1	MANES_07G106000	Protein binding activities	21	ND	
XM_021757157.1	MANES_05G162600	Protein deubiquitination and modification	21, 32	ND	
32 dpi					
XM_021736302.1	MANES_14G075900	Histone ubiquitination, transferase activity and nucleotide binding	32		ND
XM_021757031.1	MANES_05G113400	Transferase activity	32, 67		32
XM_021776057.1	MANES_13G113000	Regulation of gene expression	32, 67		21, 32
XM_021778976.1	MANES_14G062600	Nucleic acid binding and mRNA splicing, via spliceosome	32		32, 67
XM_021768457.1	MANES_09G084300	Nucleotide binding and GTPase activity	21, 32		32
XM_021741558.1	MANES_16G130700	Response to oxidative stress and peroxidase activity	21, 32, 67		21, 32, 67
XM_021757157.1	MANES_05G162600	Protein deubiquitination and peptidase activity	21, 32		32
XM_021756222.1	MANES_05G164500	Xenobiotic detoxification and transmembrane transporter activity	21, 32		32
XM_021747362.1	MANES_S092000	Phospholipid binding	32		ND
XM_021753204.1	MANES_03G180500	Electron transfer activity	ND		32, 67
XM_021768178.1	MANES_15G171300	Integral component of membrane	32		ND
XM_021742064.1	MANES_16G098000	Integral component of membrane and proteolysis	32		21, 32, 67
XM_021769303.1	MANES_10G072500	Integral component of membrane	ND		32
67 dpi					
XM_021772597.1	MANES_11G067600	Nucleotide binding	67		ND
XM_021745843.1	MANES_18G049100	DNA-binding transcription factor activity and templated transcriptional regulation	ND		67
XM_021764416.1	MANES_08G119400	Regulation of histone H3-K9 acetylation and demethylation negative including ubiquitin protein ligase activity	ND		67
XM_021778798.1	MANES_14G074200	Transferase activity and phosphorylation	ND		67
XM_021742955.1	MANES_17G063300	Oxidoreductase activity	67		ND
XM_021760962.1	MANES_06G161500	Signal transduction and defense response	ND		67
XM_021739982.1	MANES_15G129500	Plant defense response	21, 32, 67		21, 32, 67
XM_021753388.1	MANES_04G082000	Response to auxin	67		ND
XM_021736330.1	MANES_14G009400	Jasmonic acid mediated signaling pathway	21, 32, 67		21, 32, 67
XM_021756443.1	MANES_05G024300	Abscisic acid-activated signaling pathway	21, 32, 67		21, 32, 67
XM_021776202.1	MANES_13G125800	Phosphatase activity	ND		67
XM_021755460.1	MANES_04G107100	Integral component of membrane	ND		67
XM_021747109.1	MANES_S069000	Integral component of membrane	ND		67
XM_021772597.1	MANES_11G067600	Protein refolding and heat shock protein binding	67		ND

*The functional GO term was explored by using the BioMart platform through the Ensembl Plant Genes database v56 based on *Manihot esculenta* genes

**ND = Not determined or not detected by RNA-seq

**Table 2:** Fifty differentially expressed genes of SLCMV-infected KU 50 and R 11 cultivars at 32- vs. 67 dpi and RT-qPCR results (2^−∆Cq^ values)

NCBI accession numbers	Gene stable ID	GO terms*	KU50				R11			
			Log2 fold		2-∆Cq		Log2 fold		2-∆Cq	
			32 dpi	67 dpi	32 dpi	67 dpi	32 dpi	67 dpi	32 dpi	67 dpi
Epigenetic modification										
XM_021774405.1	MANES_12G090900	Acetyltransferase and transferase activity	ND**	0.232	3.48	0.41	0.345	-4.582	0.7	0.08
XM_021744159.1	MANES_17G027500	Chromatin and methylated histone binding	0.051	-0.259	3.14	0.8	-0.028	0.432	0.51	0.05
XM_021742046.1	MANES_16G100800	Histone and chromatin binding activities	ND	-0.686	5.31	0.63	-0.513	1.623	0.87	0.07
XM_021764416.1	MANES_08G119400	Histone H3-K9 demethylation, transcription, and histone binding activities	ND	ND	7.54	0.33	ND	4.625	1.72	0.12
XM_021736302.1	MANES_14G075900	Histone ubiquitination, transferase and nucleotide binding activities	3.412	-3.128	2.85	1.73	-0.823	ND	0.27	0.16
XM_021746231.1	MANES_18G066200	Methylation (at histone H3-K4 methylation)	-0.345	0.683	2.9	1.06	-0.239	0.238	0.99	0.09
XM_021749125.1	MANES_02G137800	Phosphorylation	0.178	-0.016	3.63	0.89	0.186	-0.044	0.48	0.09
XM_021776846.1	MANES_18G142500	Transferase activity and methylation	ND	1.276	1.1	0.77	ND	ND	1.17	0.68
XM_021741677.1	MANES_16G083500	Transferase activity and methylation	1.168	ND	3.78	0.61	0.138	ND	0.53	0.1
XM_021778798.1	MANES_14G074200	Transferase activity and phosphorylation	ND	ND	5.08	0.58	-0.823	2.625	0.82	0.06
Transcriptional and TFs activities										
XR_002489355.1	MANES_10G107100	Mitotic spindle checkpoint protein MAD1	1.826	-1.543	4.19	3.53	ND	ND	1.05	0.63
XM_021744546.1	MANES_13G140600	mRNA transcription	2.673	-0.908	1.16	0.9	0.916	-1.403	0.99	0.56
XM_021766905.1	MANES_09G130300	DNA replication	-0.242	-0.583	4.66	0.74	-0.693	0.711	0.77	0.08
XM_021742098.1	MANES_16G097900	DNA-binding transcription factor activity	ND	-0.647	2.24	0.94	ND	ND	1.09	0.05
XM_021755496.1	MANES_04G105000	DNA-binding transcription factor activity	-2.511	0.315	4.27	0.64	-1.768	-3.676	0.095	0.07
XM_021755704.1	MANES_04G052000	Regulation of DNA transcription and DNA-binding transcription factor activity	0.114	-0.028	2.52	1.37	0.049	-0.471	1.47	0.04
XM_021763698.1	MANES_08G134700	Regulation of DNA transcription and DNA-binding transcription factor activity	-0.408	-1.422	1.93	0.98	-1.151	0.182	1.1	0.29
XM_021740858.1	MANES_16G102600	Regulation of DNA transcription and DNA-binding transcription factor activity	0.272	-0.21	3.49	1.08	0.592	-0.362	0.26	0.18
XM_021756611.1	MANES_05G084300	Regulation of DNA transcription and DNA-binding transcription factor activity	-0.328	ND	1.96	1.14	0.213	ND	0.51	0.07
XM_021748021.1	MANES_02G191900	Regulation of DNA transcription, DNA binding activity	1.829	-0.581	3.69	0.72	ND	1.144	1	0.07
XM_021740451.1	MANES_16G043500	Regulation of DNA transcription and DNA-binding transcription factor activity	-2.511	1.21	4.4	0.61	-1.598	-2.261	0.71	0.08
XM_021778976.1	MANES_14G062600	RNA binding activities	2.777	-1.171	7.07	0.42	-0.32	-3.676	0.55	0.06
XM_021764724.1	MANES_08G040900	Nuclear-transcribed mRNA catabolic process	1.83	-0.582	5.16	0.7	ND	ND	1.77	0.02
Plant defense and oxidative activities										
XM_021742955.1	MANES_17G063300	Oxidoreductase activity	0.932	4.018	3.66	0.71	ND	ND	0.62	0.07
XM_021771507.1	MANES_11G117500	Response to oxidative stress	ND	0.232	2.99	2.03	0.176	0.44	0.75	0.1
XM_021739982.1	MANES_15G129500	Plant defense response	ND	0.315	1.49	0.86	2.23	0.51	0.85	0.277
XM_021760962.1	MANES_06G161500	Signal transduction and plant defense response	0.932	-0.647	2.93	1.06	ND	3.625	0.9	1.09
XM_021746730.1	MANES_S035000	Plant defense response	-0.992	ND	3.9	0.87	ND	ND	1.05	0.06
XM_021740858.1	MANES_16G130700	Response to oxidative stress	0.272	-0.21	3.49	1.08	0.592	-0.362	0.26	0.18
Gene expression										
XM_021758264.1	MANES_05G055700	Regulation of gene expression and RNA splicing	0.03	-0.016	1.38	9.03	-0.343	0.55	0.13	0.91
XM_021776058.1	MANES_13G113000	Regulation of gene expression	-2.473	ND	3.19	0.86	ND	ND	1.2	0.07
XM_021776057.1	MANES_13G113000	Regulation of gene expression	2.828	-0.101	2.71	1.44	-0.183	0.921	0.93	0.05
Hormones and metabolic mediated pathways										
XM_021768946.1	MANES_10G142900	Regulation of primary metabolic process	-0.03	0.239	3	0.77	0.069	-0.076	0.66	0.1
XM_021753388.1	MANES_04G082000	Response to auxin	1.826	3.122	3.68	0.67	2.58	-2.259	1.75	0.04
XM_021755537.1	MANES_04G080300	Response to auxin	-0.692	0.899	3.22	2.19	0.633	-0.128	7.17	0.06
XM_021736330.1	MANES_14G009400	Auxin-activated and jasmonic acid mediated signaling pathway	0.226	0.165	3.35	0.75	0.479	0.017	0.36	0.08
XM_021756611.1	MANES_05G084300	Auxin-activated signaling pathway	-0.328	ND	1.96	1.14	0.213	ND	0.51	0.07
XM_021740451.1	MANES_16G043500	Brassinosteroid mediated signaling pathway	-2.511	1.21	4.4	0.61	-1.598	-2.261	0.71	0.08
Translation and translational initiation										
XM_021747950.1	MANES_02G141800	Translation activities	0.3	0.025	3.61	0.82	-0.006	-0.422	0.87	0.05
XM_021779209.1	MANES_01G114900	Translation and translational initiation activities	-0.345	-0.785	4.9	0.68	-0.711	-2.996	0.92	0.07
XM_021763093.1	MANES_07G025200	Translation initiation factor activity, translational initiation, translation, and ribosome binding activities	ND	ND	5.58	0.58	-0.823	3.626	0.82	0.08

* The GO terms that performed in this table was from the further functional studied by using AmiGo and GO-CAM platforms

** ND = Not Determined or not detected by RNA-seq

At 21 dpi, there were no epigenetic modification genes in either KU 50 (tolerant) or R 11 (susceptible). However, in KU 50, there were genes related to the transcription and TF activities category solely at 21 dpi, including XM_021750310.1 (DNA and RNA binding, including TF activity function), XM_021776058.1 (regulates gene expression), and XM_021755496.1 (TF binding activity and DNA transcription). In R 11, the lack of expression of the genes XM_021750310.1 and XM_021776058.1 at 21 dpi and the other time points suggest that these genes may be silenced, possibly via TGS. Blocking specific TF binding sites may lead to the occurrence of untranscribed genes. TFs are instrumental in regulating networks of genes during viral infections and their activities can underlie resistance, tolerance, and susceptibility phenotypes in different cultivars [20]. TFs can function as either repressors or activators/enhancers, depending on their ability to bind to specific DNA sites and guide or inhibit transcription processes [29, 30]. Therefore, TFs play a crucial role in regulating gene expression by controlling the on/off switches. In this study, the TF-controlled target gene XM_021755496.1 (TF binding activity and DNA transcription) was expressed solely at 21 dpi in KU 50 (tolerant), but at either 21- or 32 dpi in R 11 (susceptible) (Table 1). This reflects that TFs are activated to control target gene expression in response to viral infection. Another gene with demonstrated DNA and TF binding activities is the brassinosteroid signaling pathway gene XM_021740451.1, which was universally detected at 21 dpi in both cultivars, but was only detected in R 11 at 32 dpi (Table 1).

The phenotypic variations and differential infection stages were combined to simplify the reflection of plant response efficiency against pathogen infection, as mentioned previously in the definition of a tolerant cultivar, because at 32 dpi (middle/recovery stage), there are neither reductions in symptom severity nor lowering of viral particles accumulation in KU 50 (tolerant) [20, 21, 31]. Hence, several genes (such as XM_021755496.1 and XM_021748021.1), which are TFs, may be involved in alterations that lead to the transcription of target genes that aid SLCMV infection, particularly in KU 50 (tolerant). However, further research is necessary to confirm this assumption.

At 32 dpi (middle/recovery stage), the gene XM_021736302.1 (which is involved in epigenetic modification, histone ubiquitination, transferase activity, and nucleotide binding mechanism) was expressed in KU 50. This suggested that XM_021736302.1 may have a role in the recovery mechanism against viral infection, especially in the KU 50 (tolerant) cultivar. This finding supports the hypothesis that the plant possesses a recovery mechanism.

Epigenetic modifications, including DNA methylation, histone modifications, and RNA-mediated gene silencing, are involved in plant adaptation to biotic stress [32, 33]. Recently, Sun et al. (2022) [33] conducted a transcriptomic analysis of DNA methylation-based adaptation to abiotic stress in plants and found DNA methylation of drought-responsive genes, demonstrating the role of DNA methylation in establishing short-term memory in stressed plants by controlling transcription. In *Potato virus Y*-infected tobacco (*Nicotiana tabacum* L.), PTGS, which can cause a continuous reduction in virus titers during the late stage of infection, is associated with recovery symptoms, and similar findings were observed in SACMV-infected cassava, suggestive of decreasing virus titers at the recovery stage [14, 16]. In tolerant cultivars such as KU 50, the TGS and PTGS pathways may serve as plant mechanisms against viral infection by altering DNA methylation and transcriptional control, leading to adaptation and activation of defense mechanisms.

At 67 dpi (late infection/late recovery stage), we observed the expression of genes related to oxidoreductase activity, signal transduction, and plant defense responses that were not expressed at 21 or 32 dpi (Table 1). For example, the gene XM_021742955.1 (which is involved in oxidoreductase activity) in KU 50 was expressed solely at 67 dpi. Several enzymes, such as peroxidase, reductase, dehydrogenase, oxidase, and hydroxylase [34], are involved in oxidoreductase activity in cells, and this activity can be facilitated by cofactors like nicotinamide adenine dinucleotides (such as NAD^+^/NADH) and flavines (such as FAD/FADH2) [35]. A nuclear-encoded chloroplastic ferredoxin–NADP^+^ oxidoreductase gene was down-regulated in *Nicotiana benthamiana* infected with *Bamboo mosaic virus* (BaMV), which increased BaMV accumulation [36]. The lack of expression of this gene may have a driving role in the development of chlorosis/mosaic symptoms during viral infection by disrupting chloroplast function, which is essential for photosynthesis.

The gene XM_021760962.1 (associated with signal transduction and plant defense response) was expressed at 67 dpi in R 11 (susceptible) but not in KU 50 (tolerant), suggesting different defense mechanisms against viral infection between the two cultivars. In addition, at 67 dpi, several epigenetic modification genes (which decrease histone H3-K9 acetylation and demethylation and exhibit ubiquitin protein ligase activity, transferase activity, and phosphorylation activity) were expressed. Two of these genes were expressed in R 11 (susceptible) but not KU 50 (tolerant), potentially indicating the distinct strategies of the two cultivars to combat viral infection.

Furthermore, at all examined time points in R 11 and KU 50 cultivars, there was a gene related to the salicylic acid-mediated signaling pathway (XM_021737402.1) and a gene that assists in responding to oxidative stress and peroxidase activity (XM_021741558.1). These genes may be housekeeping genes or common constitutively expressed genes.

Additionally, the number of deposited genes was restricted owing to the limited nature of the *Manihot esculenta* v.6 database accessed through the BioMart tool. In summary, these findings regarding plant tolerance/susceptibility contribute to our understanding of the roles of various genes in plant defense mechanisms, including immune responses and related pathways.

GO analysis revealed a potential histone modification mechanism through XM_021764416.1, which was uniquely detected in R 11 at 67 dpi (Table 1). This gene was defined as being related to histone H3-K9 acetylation and demethylation. H3-K9 acetylation is located on the histone 3 protein (H3) of chromatin and close to the transcription start site of target expressed genes, including sites where TFs bind to specific DNA sequences [37, 38].

**Fig. 3:**
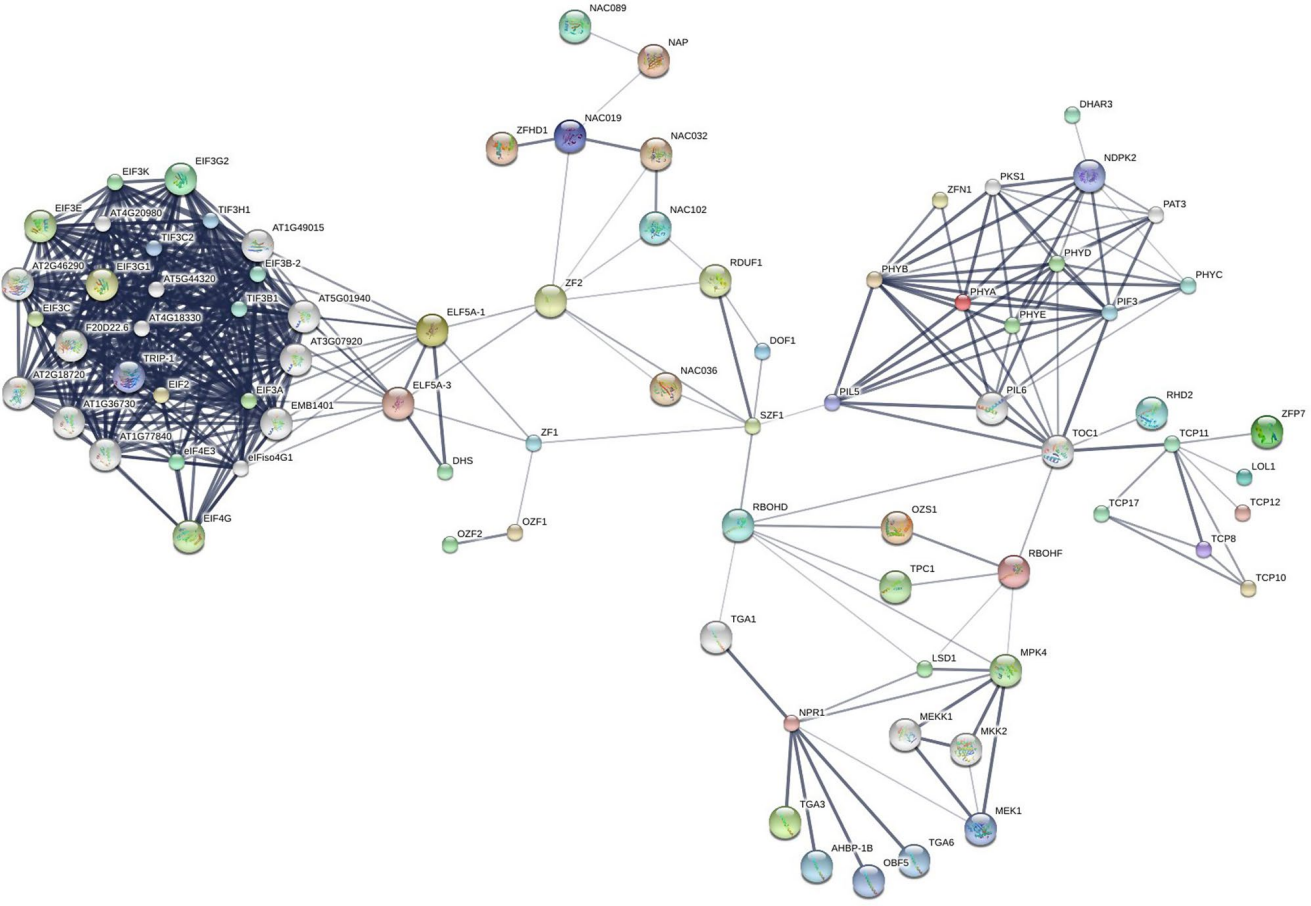
STITCH5 analysis, using a sorting parameter of the model plant *Arabidopsis thaliana* database, to construct an interaction network of gene predicted products (or protein–protein network) and their relationships with chemicals in various pathways

Subsequently, STITCH5 protein–protein interaction network analysis [39] was conducted. The STITCH5 network can help to construct, compare, and identify key nodes (predicted genes products or proteins) and pathways in biological networks, including protein–protein interaction networks, gene co-expression networks, and functional interaction networks. This is useful for understanding how different biological conditions/treatments affect network structures. Our STITCH5 analysis indicated that the XM_021764416.1 and XM_021778798.1 genes (both up-regulated at 67 dpi in R 11) were linked to the JMJ25 and TCP proteins, respectively. JMJ25 affects DNA methylation accumulation in the dark, up-regulates anthocyanin biosynthesis genes, responds to JMJ24 by inhibiting histone H3 lysine 9 methylation (H3K9me2), and cooperates with siRNA to silence genes based on RNA interference (RNAi) pathways [40, 41]. TCP proteins are involved in several metabolite biosynthesis pathways (such as those for brassinosteroid, jasmonic acid, and flavonoids), bind DNA to regulate gene expression in a manner dependent on redox status, and regulate plant development and defense responses (triggering ETI and developmental regulators that fine-tune defense signaling) [42, 43]. As shown in the visualization of the STITCH5 analysis in Fig. 3, TCP proteins were directly linked to the TOC1 protein network, which may be involved in the regulation of RBOHD, PIL5, PIL6, PIF3, PHYA, PHYB, PHYE, PHYD, RBOHF, and RHD2 proteins. RBOHF and RHD2 contribute to reactive oxygen species (ROS) production during pathogen infections [44, 45] and help to regulate the hypersensitive response involved in abscisic acid-induced stomatal closing and abscisic acid hormone, as assumed that this hormone was reinforced in an intermediated signaling of the ROS-dependent pathway [46, 47].

### DEGs in the SLCMV-infected KU 50 and R 11 cultivars at 32 dpi vs. 67 dpi

**Fig. 4:**
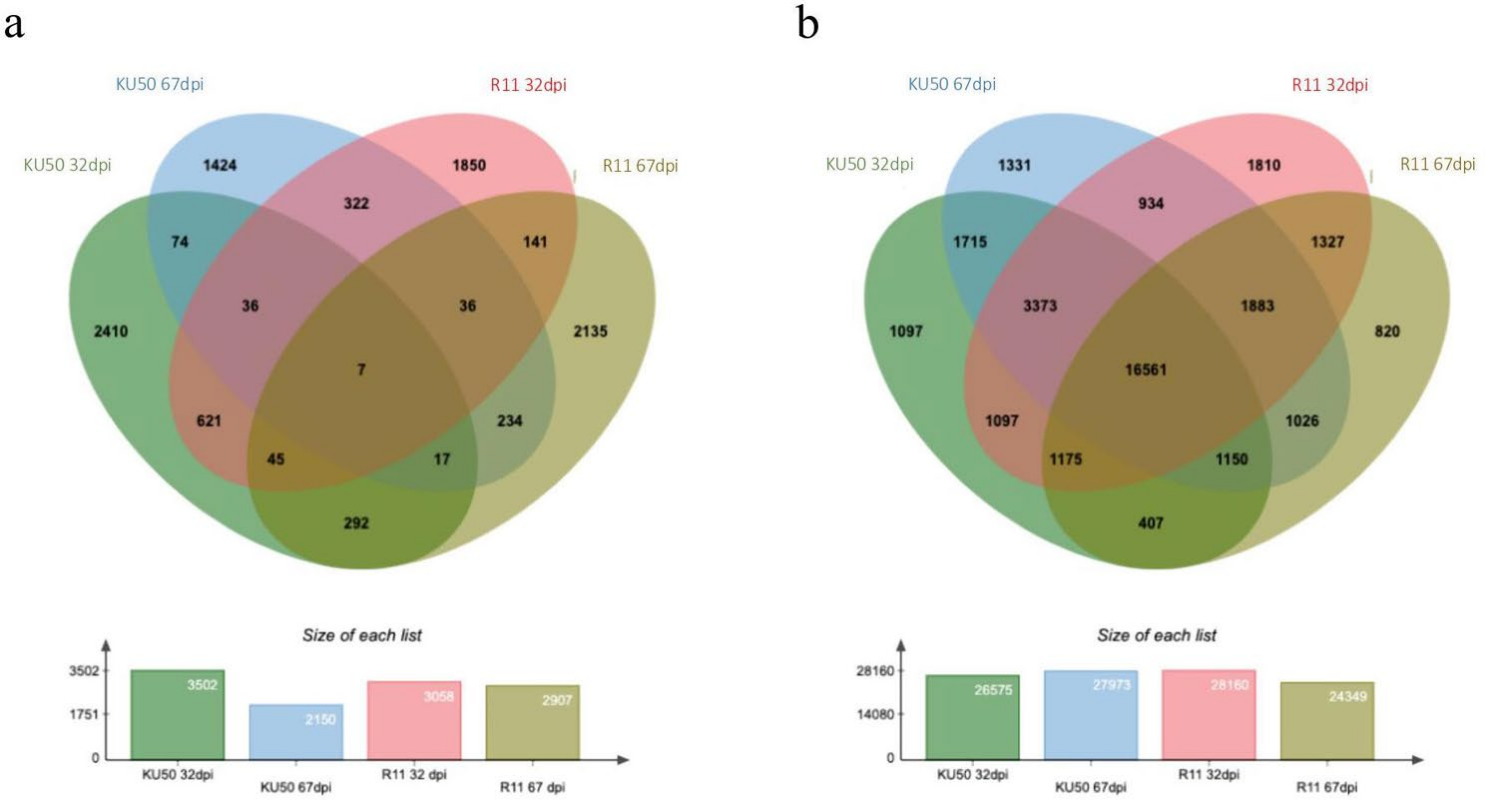
Venn diagrams of (**a**) up-regulated and (**b**) down-regulated differentially expressed genes (adjusted log_2_(fold change) at 1.5 and *p* < 0.01) in SLCMV-infected KU 50 and R 11 cultivars at 32- and 67 dpi

The RNA-seq data were quantified and genes with an adjusted log_2_(fold change) of 1.5 and *p* < 0.01 in SLCMV-infected KU 50 and R 11 cultivars at 32 dpi vs. 67 dpi were identified (Additional file 3). Venn diagrams of up- and down-regulated DEGs are shown in Fig. 4a and b, respectively. In KU 50, a total of 2,410 DEGs were up-regulated solely at 32 dpi, 1,424 genes solely at 67 dpi, and 74 genes at both time points; 1,097 DEGs were down-regulated solely at 32 dpi, 1,331 genes solely at 67 dpi, and 1,715 genes at both time points. In R 11, 1,850 genes were up-regulated solely at 32 dpi, 2,135 genes solely at 67 dpi, and 141 genes at both time points; 1,810 DEGs were down-regulated solely at 32 dpi, 820 genes solely at 67 dpi, and 1,327 genes at both time points. The Venn diagrams show that only seven genes were consistently up-regulated across all conditions (in both KU 50 and R 11 at 32 and 67 dpi), whereas 16,561 genes were consistently down-regulated (Fig. 4a–b). The consistently neither up- nor down-regulated genes across all conditions may be common/housekeeping genes. In contrast, the DEGs that were up- and down-regulated solely at 32 dpi (middle/recovery stage of SLCMV infection) may be specific genes that underlie tolerance/susceptibility. The proportion of these genes that are expressed may determine tolerance/susceptibility.

The RNA-seq data highlighted the plant defense responses to viral infection, specifically the PTGS and TGS pathways. Analyzing this dataset can help to unravel the molecular puzzle and provide a deeper understanding of how plants defend themselves against viral infections. A total of 50 DEGs of interest were selected from the RNA-seq dataset based on their GO terms (Table 1). These DEGs were classified into six categories (Table 2): (1) epigenetic modification, (2) transcription and TF activities, (3) plant defense and oxidative stress response, (4) gene expression, (5) hormone- and metabolic-mediated pathways, and (6) translation and translational initiation.

According to Table 2, in KU 50, a total of 9 DEGs were up-regulated at 32 and 67 dpi, comprising 7 genes solely at 32 dpi (XM_021776057.1, XM_021748021.1, XM_021744546.1, XM_021778976.1, XM_021736302.1, XM_021764724.1, and XR_002489355.1), 1 gene solely at 67 dpi (XM_021742955.1), and 1 gene at both 32 and 67 dpi (XM_021753388.1). Furthermore, in KU 50, 34 DEGs were down-regulated at 32 and 67 dpi, including 4 genes solely at 32 dpi (XM_021746730.1, XM_021776058.1, XM_021741677.1, and XM_021756611.1), and 12 DEGs solely at 67 dpi (XM_021776057.1, XM_021739982.1, XM_021748021.1, XM_021742046.1, XM_021744546.1, XM_021771507.1, XM_021778976.1, XM_021736302.1, XM_021764724.1, XR_002489355.1, XM_021776846.1, and XM_021742098.1). Furthermore, the remaining 18 genes were down-regulated at both 32 and 67 dpi (Table 2).

In R 11, 7 DEGs were up-regulated at 32 and 67 dpi, comprising 2 genes solely at 32 dpi (XM_021739982.1 and XM_021753388.1) and 5 genes solely at 67 dpi (XM_021742046.1, XM_021760962.1, XM_021764416.1, XM_021778798.1, and XM_021763093.1); no overlapping genes were detected at these two time points. Furthermore, in R 11, 31 DEGs were down-regulated at 32 and 67 dpi (Table 2).

Several DEGs categorized in the epigenetic modification group were specifically identified as related genes with transferase activity and methylation, and transferase activity and phosphorylation functions; these methylation and phosphorylation functions are known to occur in histone modification. The gene XM_021776846.1 (epigenetic modification category) was expressed at 67 dpi in KU 50 but not R 11. Histone methyltransferases or transferase function as either gene activators or repressors by controlling methylation, which affects DNA accessibility to TFs and thereby affects DNA transcription (in contrast, DNA methylation directly modifies the DNA sequence). This epigenetic process is crucial in the responses of organisms to environmental factors [48, 49]. Epigenetic mechanisms regulate the immune response to pathogens in plants. DNA methylation plays a key role in determining susceptibility to pathogens. Mulaudzi et al. (2023) [50] explored *Tomato curly stunt virus* (ToCSV) infections in tolerant and susceptible cultivars. Lower virus accumulations or titers in both cultivars were associated with inhibition of transcription of ToCSV genes. In addition, the tolerant cultivars (which lacked symptoms) harbored ToCSV with distinct DNA methylation patterns, whereas the susceptible cultivars harbored ToCSV with increased DNA methylation, which may hinder the activation of viral replication. Our results support the findings of Mulaudzi et al. (2023) [50] that methylation of ToCSV DNA in tolerant cultivars is involved in symptom reduction and viral replication inhibition.

The gene XM_021756611.1 (which regulates DNA-templated transcription and participates in the mitotic spindle checkpoint, binding of TFs, and the catabolic process) was expressed in both cultivars at 32 dpi and 67 dpi but was up-regulated in KU 50 vs. R 11 at 67 dpi. The STITCH5 protein–protein network analysis (Fig. 3) revealed that the gene product was associated with NAC domain proteins. The STITCH5 network also indicated that NAC domain proteins were often connected to zinc finger domain proteins such as zinc finger homeodomain 1 (ZFHD1), zinc finger protein 2 (ZF2), and salt-inducible zinc finger 1 (SZF1). This suggests an important relationship between NAC domain proteins and genes encoding zinc finger domain proteins. Zinc finger and NAC domain proteins coordinate through the PTI and ETI pathways [51–53]. In transgenic *Arabidopsis thaliana*, a zinc finger domain protein (ZFHD1) was co-expressed with NAC domain protein [54]. Both zinc finger and NAC domain proteins play a role in reprogrammed cell death in the root cap of *Arabidopsis thaliana*, with up-regulation of zinc finger and NAC domain proteins being indirectly mediated by SOMBRERO (SMB), an NAC domain TF [55].

Genes in the plant defense category were mostly involved in reactive oxygen species, oxidoreductase activity, signal transduction, oxidative stress responses, and basic defense responses. Analysis of the DEGs in this category revealed that two genes (XM_021771507.1 and XM_021739982.1) were not expressed at 32 dpi in KU 50 (tolerant), but were expressed at 67 dpi in KU 50 and at both time points in R 11. Additionally, one gene (XM_021739982.1; helps to modify metabolic and enzymatic activities during various abiotic/biotic stresses) was up-regulated solely at 32 dpi in R 11 (susceptible). The accumulation of ROS due to oxidative stress can have a negative effect on proteins and DNA, ultimately destroying plant cells [56, 57]. Several virus species induce oxidative stress to facilitate viral replication/colonization of the whole plant, as oxidative stress can assist the viral infection. Plant mitochondria are the source of oxidative responses and phosphorylation. They are also a key source of ROS during viral infections, as viral proteins interact with mitochondrial membranes and other mitochondria-associated components, leading to an eventual increase in ROS. As mitochondria are instrumental in plant energy production (via oxidative phosphorylation), increasing mitochondrial ROS production may lead to a plant respiratory disorder [58, 59]. During a viral infection, the up-regulation of oxidative phosphorylation genes in plants may be indicative of increased viral titers that are enhancing the interaction of viral proteins and mitochondrial membranes and leading to ROS production. Such ROS products can nonspecifically damage plant cells and cause oxidative stress-induced plant programmed cell death [59, 60]. Király et al. (2021) [61] assessed the defense responses to *Potato virus X* (PVX) in tobacco (*Nicotiana tabacum*) by studying symptomless plants with extreme resistance to PVX and plants with a hypersensitive response-type resistance to *Tobacco mosaic virus* (mediated by the *Rx1* resistance gene). They reported that treatment of PVX-susceptible plants with ROS (superoxide)-generating agents led to hypersensitive response-related defense mechanisms (such as ROS-regulator gene up-regulation, increased antioxidants, and programmed cell death). This resulted in hypersensitive response-like symptoms and decreased PVX titers and indicated that ROS accumulation may inhibit PVX replication in the plants with extreme resistance. Mohammadi et al. (2021) [62] reviewed the effects of ROS and oxidative stress during fungal infections in several crops such as *Arabidopsis*, potatoes, and tomatoes, reporting that higher ROS accumulation was promoted plant programmed cell death in infected tissues and restricted the viral infection, because of the high toxicity. To conclude, we found that the higher ROS accumulation was observed in this study as reflected as term of the ROS related gene function such as XM_021739982.1, this gene may have a potential role in plant defense responses to viral infection, particularly in susceptible cultivars.

### RT-qPCR validation of selected DEGs

The expression of 50 selected DEGs was assessed using RT-qPCR with specific primers. The RNA-seq and RT-qPCR data indicated that there were discrepancies in gene expression across different conditions (such as KU 50 and R 11 at 32 and 67 dpi). These results may be attributable to the filtering based on the *p*-value in the RNA-seq analysis of DEGs, as some genes did not attain the selection criterion of *p* < 0.01 and were not included in the results, although they were detected by RT-qPCR. These genes are displayed in Table 2 on the RNA-seq results as “ND” for “Not Determined or Not Detected by RNA-seq”.

A total of 20 genes were not detected by RNA-seq but were detected by RT-qPCR based on 2^−∆Cq^ values. Weakly expressed genes are difficult to detect by RNA-seq, which may be why some DEGs were not identified by RNA-seq and not annotated. Nevertheless, nine key genes (XM_021764416.1, XM_021736302.1, XM_021741677.1, XM_021744546.1, XM_021778976.1, XM_021739982.1, XM_021776057.1, XM_021753388.1, and XM_021763093.1) that were relevant to the study objective were validated by comparing the RNA-seq and RT-qPCR data on gene expression patterns, based on adjusted log_2_(fold change) and 2^−∆Cq^ values, respectively. This information is valuable for identifying marker genes that are potentially related to defense against SLCMV in cassava. These findings may benefit future research in this field.

Table 2 shows that among the 50 selected DEGs, up-regulated DEGs were rarer than down-regulated DEGs. KU 50 had more up-regulated DEGs than R 11, and down-regulated DEGs were more common in KU 50 than in R 11.

The gene XM_021741677.1 (epigenetic modification category; associated with transferase activity and methylation) was down-regulated solely at 32 dpi in KU 50, based on RNA-seq. This was consistent with its peak RT-qPCR expression (2^−∆Cq^ value) in KU 50 at 32 dpi. XM_021736302.1 (the epigenetic modification category has a crucial role in histone ubiquitination, transferase activity, and nucleotide binding) was up-regulated in KU 50 at 32 dpi, based on RNA-seq. Likewise, RT-qPCR showed peak expression of this gene in KU 50 at 32 dpi. The genes XM_021744546.1 (transcription and TF activities category; associated with mRNA transcription) and XM_021746730.1 (plant defense and oxidative stress response category; has a role in the plant defense response) had peak RT-qPCR (2^−∆Cq^ values) in KU 50 at 32 dpi. These genes were up- and down-regulated, respectively, solely at 32 dpi in KU 50, based on RNA-seq. The four genes mentioned here, which were all down- or up-regulated in the middle/recovery stage of the infection, may help to regulate the viral defense response at 32 dpi in the tolerant cultivar KU 50. Further analysis of these genes at each time point could provide valuable insights into their functional roles and facilitate understanding of the molecular mechanisms underlying SLCMV defense mechanisms in KU 50.

## Conclusions

Gene expression, including differential gene expression, influences the plant immune response to viral infections. Our transcriptomic analysis using next-generation sequencing identified DEGs in susceptible (R 11) and tolerant (KU 50) cultivars of cassava. Gene expression was altered during SLCMV infection, and these genes were potentially associated with stress responses, including immunity, hormone regulation, and metabolite changes. We also found that specific genes were expressed at specific time points, underlying the phenotypic variation between the different cultivars at different time points. Furthermore, we found some up-regulated DEGs (XM_021736302.1, XM_021744546.1, and XM_021741677.1) that were involved in epigenetic modification (transferase activity and methylation, believed to function in the TGS pathway) in KU 50 at 32 dpi, which is recognized as the recovery stage of SLCMV infection. Additionally, other DEGs between 32 and 67 dpi were involved in PTGS, reprogrammed cell death, hormone regulation, and metabolite changes. Thus, our findings regarding this case study of a pathogenic viral infection in plants highlight the pivotal role of gene transcription regulation and its association with plant phenotypic variation. We demonstrated that during the middle/recovery stage of infection (32 dpi), PTGS and TGS genes were expressed in KU 50. This indicates that plant defense responses occurred during this stage, which helped reduce disease symptoms in this tolerant cultivar. These findings provide insights into the DEG profiles in cassava cultivars during SLCMV infection, gene regulatory networks, and the mechanisms that activate or inhibit gene transcription and plant responses. Moreover, some of the results and basic knowledge obtained in this research could be beneficial for guiding and assisting other virologists, cassava breeders, plant pathologists, and further interested researchers who study plant–virus defense mechanisms (especially in the case of SLCMV-infected cassava) and molecular markers. In addition, our findings could contribute to future strategies to reduce and prevent the spread of SLCMV as well as increasing understanding of plant–virus interactions.

## Methods

### Plant material preparation

Healthy and SLCMV-infected stems from the tolerant Kasetsart 50 (KU 50) and the susceptible Rayong 11 (R 11) cassava cultivars were sourced from the Thai Tapioca Development Institute. The stems were prepared by cutting them into lengths of 13–15 cm, with three or four buds remaining on each cut piece. Subsequently, these stems were cultivated in a greenhouse of the Department of Plant Pathology at Kasetsart University (Bangkok, Thailand). This controlled environment provided suitable conditions for studying cassava responses to SLCMV infection.

### SLCMV inoculation

The grafting procedure outlined by Hemniam et al. (2019) [63] was employed for SLCMV inoculation. In this process, 2-month-old SLCMV-infected cassava plants were used as rootstocks, while 2-month-old healthy cassava stems served as scions. There were three replicates per cultivar per time point. To ensure that the scions were disease-free, PCR was conducted as described by Saokham et al. (2021) [10] to detect SLCMV in KU 50 and R 11. The grafted plants were maintained in a greenhouse at 25–29 °C and a relative humidity of 70–80%. Two leaves were retained on each rootstock until new leaves developed, typically at around 20 days after the grafting procedure. Leaves were collected at three time points (21, 32, and 67 dpi, equivalent to days after the grafting procedure) and were promptly placed in liquid nitrogen and stored at − 80 °C until further use.

### DNA extraction and PCR-based confirmation of SLCMV infection

Total DNA of the leaves was extracted using a modified cetyltrimethylammonium bromide (CTAB) method [64]. DNA quantity and quality were assessed using gel electrophoresis. A 1.5% agarose gel was prepared using 0.5× tris-acetate-ethylenediaminetetraacetic acid (EDTA) (TAE) buffer (1 M Tris-HCl [pH 8], 0.5 M EDTA, and glacial acetic acid). The DNA samples, along with a 1-kb DNA ladder (Thermo Fisher Scientific; Waltham, MA, USA) as a reference, were loaded onto the gel and electrophoresis was conducted at 100 V for 30 min. The results were visualized using SYNGENE software (Synoptics Ltd.; Cambridge, UK). DNA quantity and purity were then assessed using a NanoDrop® ND-1000 spectrophotometer (Thermo Fisher Scientific).

The PCR technique to detect SLCMV *AV1* gene fragments followed the method described by Saokham et al. (2021) [10] using forward (5′-GTT GAA GGT ACT TAT TCC C-3′) and reverse (5′-TAT TAA TAC GGT TGT AAA CGC-3′) primers.

### RNA extraction

The collected leaves that had been stored at − 80 °C were subsequently used for RNA extraction, which was performed following the mini-scale protocol described by Behnam et al. (2018) [65], with slight modifications. Thereafter, RNA quantity and purity were assessed using a NanoDrop® ND-1000 spectrophotometer. The NanoDrop thresholds of 1.8–2.2 for OD260/280 and ≥ 1.8 for OD260/230 indicate purity. RNA quality was also assessed using electrophoresis on a 1.5% agarose gel with RedSafe Nucleic Acid Staining Solution (iNtRON Biotechnology; Sangdaewon, South Korea) in 0.5× TAE buffer (1 M Tris-HCl [pH 8], 0.5 M EDTA, and glacial acetic acid) at 100 V for 30 min. A 1-kb DNA ladder (Thermo Fisher Scientific) was used as the reference. The results were visualized using SYNGENE^®^ software (Synoptics Ltd).

### Library preparation, RNA sequencing, and data analysis

Library preparation and paired-end 150-bp RNA sequencing were performed by Novogene Co. Ltd. (Beijing, China) using Illumina NovaSeq 6000 platforms. The raw sequencing FASTQ data were mapped using the *Manihot esculenta*_v8 transcript database and then submitted to the NCBI (https://www.ncbi.nlm.nih.gov/) Sequence Read Archive under RefSeq assembly accession GCF_001659605.2.

The Salmon v.1.10.1 bioinformatic tool was used to quantify the transcript levels of genes in each cultivar at each time point [66]. A Python v.3.11 custom script was used to manipulate the quantitative files to produce a table of the transcript levels [67]. Differential expression analysis was then conducted using DESeq2 in R v.4.1.2 to identify DEGs (*p* < 0.01 and adjusted log_2_(fold change) at 1.5) between SLCMV-infected KU 50 vs. R 11 cultivars at (i) an early to middle stage of SLCMV infection (21 to 32 dpi), and (ii) a middle to late stage of SLCMV infection (32 to 67 dpi).

### Annotation of DEGs in SLCMV-infected KU 50 and R 11

Venn diagrams were created using the jvenn platform (http://jvenn.toulouse.inra.fr/app/index.html) to visualize the distribution of all transcripts or genes in SLCMV-infected (i) KU 50 and (ii) R 11 at 21, 32, and 67 dpi and the distribution of (i) up-regulated and (ii) down-regulated DEGs in SLCMV-infected KU 50 and R 11 at 32 dpi vs. 67 dpi [68]. NCBI accession numbers (representing transcript or gene IDs) were determined based on *Manihot esculenta* genes (genome assembly *Manihot esculenta* v6) by using the BioMart platform to access the Ensembl Plant Genes database v56 [69].

Next, for each DEG, the following information was obtained using AmiGo2 (http://amigo.geneontology.org/amigo, accessed on June 1, 2023): GO annotations and their corresponding GO identifiers, gene descriptions, and transcript and gene stable IDs. The GO terms for each gene were further studied using AmiGo [70] and GO-CAM platforms [71] (http://geneontology.org, accessed on June 1, 2023). The PANTHER 17.0 database (http://go.pantherdb.org, accessed on June 1, 2023) was used for Panther classification identification and other information. Homolog function information based on the model plant *Arabidopsis thaliana*-related gene ontology was obtained from The *Arabidopsis* Information Resource (TAIR) (www.arabidopsis.org, accessed on June 1, 2023). STITCH v.5 was used as an analysis tool for illustrating and estimating protein–protein interactions based on predictions of linked chemical participants [39].

### RT-qPCR validation

A cDNA library was constructed based on the total RNA in each cultivar at each time point using reverse transcriptase (Thermo Fisher Scientific). The total RNA samples were the same as those used for RNA-seq analysis. Next, 50 genes of interest were selected for RT-qPCR validation based on RNA-seq data parameters of *p* < 0.01 and adjusted log_2_(fold change) at 1.5 (with ≥ 1.5 verified as up-regulated and < 1.5 indicated as down-regulated). These selected genes were associated with (1) epigenetic modification, (2) transcription and TF activities, (3) plant defense and oxidative stress response, (4) gene expression, (5) hormone- and metabolite-related activities, and (6) translation and translational initiation activities, according to GO analysis.

Primers were designed for the 50 target genes using the online Primer3 and BLAST programs [72], available via the NCBI tool. This was based on the NCBI accession numbers obtained by BLAST searches involving each of the target transcripts (Additional file 1). For RT-qPCR, 0.5 µL of forward primer, 0.5 µL of reverse primer, 3 µL of nuclease-free water, and 1 µL of cDNA as a template (after adjustment of the cDNA concentration to 100 ng/mL in all samples) were mixed together, along with 5 µL of qPCRBIO 100× SyGreen Mix Lo-ROX (COPENHAGEN BIOTECH SUPPLY; Denmark). The mixture was subjected to RT-qPCR using a CFX96 Real-Time PCR Detection System (Bio-Rad; CA, USA). ∆Cq was calculated as “Cq of normalized target samples” – “Cq of normalized control samples”, and then 2^−∆Cq^ was calculated to quantify the relative gene expression between the pairs of groups, that is, between SLCMV-infected R 11 (susceptible) vs. KU 50 (tolerant) at (i) 32 dpi vs. (ii) 67 dpi.

### Electronic supplementary material

Below is the link to the electronic supplementary material.

Supplementary Material 1

Supplementary Material 2

Supplementary Material 3

## Data Availability

The datasets generated and analysed in the current study are available in the NCBI database under the Sequence Read Archive (SRA) accession number PRJNA1040252 and the data sets supporting the conclusions of this article are included with the article and its additional files.
