# Supplementary material for: Comparative transcriptomics analysis reveals defense mechanisms of Manihot esculenta Crantz against Sri Lanka Cassava MosaicVirus
Source: BMC Genomics. 2024 May 2;25:436. doi: 10.1186/s12864-024-10315-0 (PMC11067156; doi:10.1186/s12864-024-10315-0)
Supplement: Supplementary file 1 — Supplementary Material 1 [file 12864_2024_10315_MOESM1_ESM.docx]

**Additional file 1** Primer sequence for Real-time RT-PCR KU50 and R11 SLCMV infection.

| **No** | **Name primer** | **Forward primer**  **5’🡪 3’** | **Reverse primer**  **5’🡪 3’** | **Tm** | **PCR product (bp)** |
| --- | --- | --- | --- | --- | --- |
| 1 | XM_021742955.1 | GTGTTCAAAAGCTGGCCGAA | AGCAACGAGCTTTCATCGGA | 60 | 100 |
| 2 | [XM_021746730.1](http://www.ncbi.nlm.nih.gov/sites/entrez?db=nuccore&cmd=search&term=XM_021746730.1) | GATGTGTGGAGTGAGGACCC | ACACGATTGCTACGAGTGGT | 60 | 104 |
| 3 | [XM_021776058.1](http://www.ncbi.nlm.nih.gov/sites/entrez?db=nuccore&cmd=search&term=XM_021776058.1) | ATTGAAAGCCCTGAAGCCCA | GGGACGGCTGAAATGAAGGA | 60 | 92 |
| 4 | [XM_021776057.1](http://www.ncbi.nlm.nih.gov/sites/entrez?db=nuccore&cmd=search&term=XM_021776057.1) | CCTTCATTTCAGCCGTCCCT | AGCGCCGTTTACAGGATTCA | 60 | 98 |
| 5 | XM_021741677.1 | TCGACATGCCAGGGTTGAAA | AGCCCCTTCCTTCTCCTCAT | 60 | 107 |
| 6 | XM_021739982.1 | TTGGTCTGGAAGGTGACGTG | CTTTTGACAAACACCCCTGACC | 60 | 90 |
| 7 | XM_021748021.1 | TCTGAACATGGACCCCCTCA | AGCTTCGTAACCTGGCCTTT | 60 | 90 |
| 8 | XM_021756611.1 | CACAAGTAGTGGGTTGGCCT | TTGCCGGATAGCTTCTGCTC | 60 | 100 |
| 9 | [XM_021746231.1](http://www.ncbi.nlm.nih.gov/sites/entrez?db=nuccore&cmd=search&term=XM_021746231.1) | GGTCTTCCTTGAGTGAGGGC | ATGGACTGCTGTTCTCCAGC | 60 | 92 |
| 10 | [XM_021747950.1](http://www.ncbi.nlm.nih.gov/sites/entrez?db=nuccore&cmd=search&term=XM_021747950.1) | GCTCTCTGGTCTGCACCATT | GCTGCAGGAGACCTTGTCTT | 60 | 110 |
| 11 | [XM_021763698.1](http://www.ncbi.nlm.nih.gov/sites/entrez?db=nuccore&cmd=search&term=XM_021763698.1) | AGCCATTTCATGGTCAGCCA | CTGTGGCTTGTCCTCGTCTT | 60 | 99 |
| 12 | [XM_021755704.1](http://www.ncbi.nlm.nih.gov/sites/entrez?db=nuccore&cmd=search&term=XM_021755704.1) | GCTGCTTCACAACCTGAACC | CCTTCCACATCCTACGCTCG | 60 | 94 |
| 13 | [XM_021742046.1](http://www.ncbi.nlm.nih.gov/sites/entrez?db=nuccore&cmd=search&term=XM_021742046.1) | AGATAACCTCAGCATGGCCG | GCTGACCAGCCAGAGAATGT | 60 | 104 |
| 14 | [XM_021760962.1](http://www.ncbi.nlm.nih.gov/sites/entrez?db=nuccore&cmd=search&term=XM_021760962.1) | AGAACCCAGATGCTCCAAGC | TCCTTGTTCGTCGGTGACAG | 60 | 95 |
| 15 | [XM_021753388.1](http://www.ncbi.nlm.nih.gov/sites/entrez?db=nuccore&cmd=search&term=XM_021753388.1) | ATAGTTCCGGTGGCGTTGTT | AGCGCCCATTGGATGATTGA | 60 | 93 |
| 16 | [XM_021764416.1](http://www.ncbi.nlm.nih.gov/sites/entrez?db=nuccore&cmd=search&term=XM_021764416.1) | AGAACCAAGGCCAAGACTCG | AGCCACCATACTCCTTTGGC | 60 | 98 |
| 17 | [XM_021736330.1](http://www.ncbi.nlm.nih.gov/sites/entrez?db=nuccore&cmd=search&term=XM_021736330.1) | TGAAGGAGCTGAGCAACGAG | TCAGCCACGGCCTGATTAAG | 60 | 100 |
| 18 | [XM_021740451.1](http://www.ncbi.nlm.nih.gov/sites/entrez?db=nuccore&cmd=search&term=XM_021740451.1) | CGTTCCCCTCATCTATGCCC | AATGTGGGTGATGACGGTCC | 60 | 118 |
| 19 | [XM_021744546.1](http://www.ncbi.nlm.nih.gov/sites/entrez?db=nuccore&cmd=search&term=XM_021744546.1) | TAGTCTCGACGCGCTAATCG | CTCACTGCTCTCGCTCCAAA | 60 | 93 |
| 20 | [XM_021744159.1](http://www.ncbi.nlm.nih.gov/sites/entrez?db=nuccore&cmd=search&term=XM_021744159.1) | GCCCAAACCTGGCAAAAGAG | TATGGTGGCTTTCCGGTGTC | 60 | 114 |
| 21 | [XM_021755496.1](http://www.ncbi.nlm.nih.gov/sites/entrez?db=nuccore&cmd=search&term=XM_021755496.1) | TGGCATTGCCGGAAAAAGTG | TTGCTGCTGGCTATTCCCAA | 60 | 94 |
| 22 | [XM_021749125.1](http://www.ncbi.nlm.nih.gov/sites/entrez?db=nuccore&cmd=search&term=XM_021749125.1) | ATACAAGGCTCGTGATCGGG | GCAGTGCTTGGAACACCTTC | 60 | 90 |
| 23 | [XM_021755537.1](http://www.ncbi.nlm.nih.gov/sites/entrez?db=nuccore&cmd=search&term=XM_021755537.1) | TCCATCTCCTCCCTTGGGTT | GTGCGCTCTACAACCTCTGT | 60 | 107 |
| 24 | [XM_021757249.1](http://www.ncbi.nlm.nih.gov/sites/entrez?db=nuccore&cmd=search&term=XM_021757249.1) | AAGCTGCATGTGACGGGATT | CCGAGGTGTTGGACCTCTTG | 60 | 96 |
| 25 | [XM_021758264.1](http://www.ncbi.nlm.nih.gov/sites/entrez?db=nuccore&cmd=search&term=XM_021758264.1) | GCAAGGAGGTCTTCCTCACC | CCAGTTGGCATCGTCTTCCT | 60 | 92 |
| 26 | [XM_021744546.1](http://www.ncbi.nlm.nih.gov/sites/entrez?db=nuccore&cmd=search&term=XM_021744546.1) | AGTCAGCATCAGGAGGGTCT | ACGATGCTCCTCTTCCTCCT | 60 | 99 |
| 27 | [XM_021766905.1](http://www.ncbi.nlm.nih.gov/sites/entrez?db=nuccore&cmd=search&term=XM_021766905.1) | GAAATCGCCCAAAGACGCAG | TTGCCTCAACGCTTCCTCAT | 60 | 121 |
| 28 | [XM_021768946.1](http://www.ncbi.nlm.nih.gov/sites/entrez?db=nuccore&cmd=search&term=XM_021768946.1) | GTTCCGCCAAAGCTGCATAG | GTTCTCACCAACGGACCCAT | 60 | 111 |
| 29 | [XM_021771507.1](http://www.ncbi.nlm.nih.gov/sites/entrez?db=nuccore&cmd=search&term=XM_021771507.1) | TTCCAAATGTGGCTTGACGC | GTTGCAAGGAAACGCCAAGA | 60 | 100 |
| 30 | [XM_021778976.1](http://www.ncbi.nlm.nih.gov/sites/entrez?db=nuccore&cmd=search&term=XM_021778976.1) | GGAGGGAGGGAAAGAAACCG | GGCATGGAAGTAGAGGCCAG | 60 | 91 |
| 31 | [XM_021779209.1](http://www.ncbi.nlm.nih.gov/sites/entrez?db=nuccore&cmd=search&term=XM_021779209.1) | TGGTGCTGCTCCTGTTCAAA | TGGACAATCACAGGTCCAGC | 60 | 105 |
| 32 | [XM_021740858.1](http://www.ncbi.nlm.nih.gov/sites/entrez?db=nuccore&cmd=search&term=XM_021740858.1) | TGGTCCTGAGATAGCAGCCA | TTCTTCCTCGAACTTGCGCT | 60 | 116 |
| 33 | XM_021736302.1 | GTGACCCCAACCCCAATTCT | GTCCAGCTCTGCTCCACAAT | 60 | 109 |
| 34 | [XM_021764724.1](http://www.ncbi.nlm.nih.gov/sites/entrez?db=nuccore&cmd=search&term=XM_021764724.1) | CAACGGAGGAGAATGGACCC | CAACAAGGACTCCACCGTCA | 60 | 123 |
| 35 | [XR_002489355.1](http://www.ncbi.nlm.nih.gov/sites/entrez?db=nuccore&cmd=search&term=XR_002489355.1) | TGTTGCTCTCTTCTCGCGTT | GCAATGCAGCACCTGTCAAG | 60 | 95 |
| 36 | XM_021776846.1 | GATTGGCAAGGCAGTGGTTG | TTGGCCATATTCCCAGTGCC | 60 | 93 |
| 37 | [XM_021778798.1](http://www.ncbi.nlm.nih.gov/sites/entrez?db=nuccore&cmd=search&term=XM_021778798.1) | CGCTTTCCCTCTGGTGCTTA | GGGCAGAGTTGTTGTGGGTA | 60 | 102 |
| 38 | [XM_021763093.1](http://www.ncbi.nlm.nih.gov/sites/entrez?db=nuccore&cmd=search&term=XM_021763093.1) | CTGCTCACCGTACTTCCTCTC | CCTGTTTTGACCCACCACCA | 60 | 133 |
| 39 | [XM_021742098.1](http://www.ncbi.nlm.nih.gov/sites/entrez?db=nuccore&cmd=search&term=XM_021742098.1) | CGTGCAAAGCTAGAGGCTCA | AGAAAGCGTCCACCAGATCC | 60 | 113 |
| 40 | [XM_021774405.1](http://www.ncbi.nlm.nih.gov/sites/entrez?db=nuccore&cmd=search&term=XM_021774405.1) | TAGAGAAGCCCAACGGAAGC | CACGATTTCTCCCACAGCCT | 60 | 99 |
| 41 | XM_021763603.2 | AAATTCGACCAGGGTTGCCA | TGTGGTGGCCGTAGTATGTG | 60 | 91 |
| 42 | XM_021741686.2 | ACCCCAAAGACAAAAGCCCA | CTTTTTGACGGGGCAACCTG | 60 | 134 |
| 43 | XM_021774985.2 | TCAAGGCCTTCCAACTTGCT | TTGACTTCGAATCAGGGCCG | 60 | 117 |
| 44 | XM_021767775.2 | TACGTCAGTTGGTTGTCCCG | ATGGTTGTGCTTCCCTTCGT | 60 | 94 |
| 45 | XM_021776299.2 | GTTTCTTCAGCGGGAAAGCC | GCAATGGCAACGACCAGAAG | 60 | 114 |
| 46 | XM_021751457.2 | GGTACTTTCGCCTTCTGCCT | TCCACAGCTGTCCTGTTAGC | 60 | 97 |
| 47 | XM_021761805.2 | GAGAGCTCGTTCAAGGCCAA | CATCAGCTGAGAGCAACCCA | 60 | 90 |
| 48 | XM_021757556.2 | CGTCGAATTTGATGACCGGC | GGAGTTCGGCTGAGCTAGAG | 60 | 102 |
| 49 | XM_021757679.2 | AGCATGTCGAAAGGGCATCA | ACCCTGAGTTGACATGGCTG | 60 | 111 |
| 50 | XM_021769159.2 | TCACCCTCAAAGCGTCACAT | AGGAAGATGGCAGGAAAGAGC | 60 | 95 |
